# Endoplasmic reticulum: a focal point of Zika virus infection

**DOI:** 10.1186/s12929-020-0618-6

**Published:** 2020-01-20

**Authors:** Muhammad Izzuddin Mohd Ropidi, Ahmad Suhail Khazali, Nurshamimi Nor Rashid, Rohana Yusof

**Affiliations:** Department of Molecular Medicine, Faculty of Medicine, University of Malaya, 50603 Kuala Lumpur, Malaysia

**Keywords:** Zika virus, Endoplasmic reticulum, Unfolded protein response, Stress granules, Reticulophagy, Paraptosis, Cytoplasmic vacuolization

## Abstract

Zika virus (ZIKV) belongs to the *Flavivirus* genus of the *Flaviviridae* family. It is an arbovirus that can cause congenital abnormalities and is sexually transmissible. A series of outbreaks accompanied by unexpected severe clinical complications have captured medical attention to further characterize the clinical features of congenital ZIKV syndrome and its underlying pathophysiological mechanisms. Endoplasmic reticulum (ER) and ER-related proteins are essential in ZIKV genome replication. This review highlights the subcellular localization of ZIKV to the ER and ZIKV modulation on the architecture of the ER. This review also discusses ZIKV interaction with ER proteins such as signal peptidase complex subunit 1 (SPCS1), ER membrane complex (EMC) subunits, and ER translocon for viral replication. Furthermore, the review covers several important resulting effects of ZIKV infection to the ER and cellular processes including ER stress, reticulophagy, and paraptosis-like death. Pharmacological targeting of ZIKV-affected ER-resident proteins and ER-associated components demonstrate promising signs of combating ZIKV infection and rescuing host organisms from severe neurologic sequelae.

## Introduction

Zika virus (ZIKV) is a mosquito-borne virus that belongs to the *Flaviviridae* family together with other notable flaviviruses such as dengue virus (DENV), West Nile virus (WNV), Japanese Encephalitis virus (JEV), and yellow fever virus (YFV). ZIKV was first isolated from a febrile rhesus monkey in April 1947 and was subsequently isolated from *Aedes africanus* mosquitoes 9 months later in Zika Forest of Uganda [1] {Dick, 1952 #15}. Despite its discovery more than a half-century ago, ZIKV received little attention due to sporadic cases of human infection with mild and self-limiting symptoms [2]. ZIKV was put under scrutiny following its first outbreak in the Yap Island of Micronesia in 2007. During this outbreak, 185 suspected cases of ZIKV infection were reported, and at least 24% of these patients were either serologically or molecularly confirmed for ZIKV infection [3]. The same study estimated that 5005 island residents (73%) were infected, of which 919 were symptomatic [3].

ZIKV-infected patients typically present mild clinical symptoms such as fever, maculopapular rash, conjunctivitis, and arthralgia [2]. Nevertheless, the second ZIKV outbreak in French Polynesia in 2013 provided the first compelling relationship between ZIKV infection and a neurological complication, where a woman was diagnosed with Guillain-Barré syndrome (GBS), an autoimmune disease typically affecting motor neuron functions, a week following the onset of ZIKV-like symptoms [4]. This epidemic also recorded a 20-fold increase of GBS incidence, where 41 GBS-diagnosed patients (98%) were serologically positive for ZIKV [2, 5].

The sudden spike of microcephaly cases among newborns in Brazil following the 2015–2016 ZIKV outbreak triggered the Brazilian Ministry of Health and the World Health Organization to declare ZIKV as a national and international public health emergency [6, 7]. The causative link between ZIKV and congenital neurological anomalies was established based on several evidence including the detection of ZIKV RNA within the amniotic fluid acquired from ZIKV-infected mothers with confirmed fetal microcephaly case [8, 9]. Subsequent clinical and pre-clinical findings further supported the causal relationship between ZIKV infection and microcephaly in newborns [10–12].

ZIKV is believed to infect cells through receptor-mediated endocytosis. These putative receptors include Cluster of Differentiation 209 (CD209), Tyrosine-protein kinase receptor Tyro3, and AXL, where overexpression of these receptors in ZIKV-impervious HEK293T cells rendered the cells susceptible to ZIKV infection [13]. In particular, the role of AXL in ZIKV infection has been extensively investigated due to its crucial role in dengue virus infection [13]. During ZIKV infection, AXL mediates ZIKV entry indirectly whereby the phosphatidylserine extension on ZIKV lipid membrane binds to Growth arrest-specific 6 (Gas6), one of the ligands for AXL that serves as a bridge for ZIKV and AXL interaction, resulting in clathrin-mediated virus internalization [14, 15]. The acidic microenvironment within the endosome promotes fusion between the virus envelope proteins and the endosomal membrane resulting in the release of ZIKV genome into the host cell cytosolic space [15, 16].

The ZIKV genome is a positive-sense, single-stranded RNA ((+)ssRNA) of approximately 11,000 bases in length [2]. ZIKV genome contains a single open reading frame that encodes three structural and seven non-structural (NS) proteins. These structural proteins consist of capsid (C), pre-membrane (prM), and envelope (Env) proteins, which are predominantly involved in viral pathogenesis and virion structure. The seven non-structural proteins, NS1, NS2A, NS2B, NS3, NS4A, NS4B, and NS5 proteins, largely contribute towards the purposes of viral pathogenesis, replication, and immune evasion [17]. ZIKV utilizes host translational machinery to produce a single polyprotein that is further cleaved by viral NS2B-NS3 serine protease and host cell protease into functional viral proteins [18]. These viral proteins are then distributed to cellular compartments for various functions [19].

## ZIKV proteins localize to distinct subcellular compartments

ZIKV proteins are primarily distributed within and in close proximity to several endomembrane compartments including the endoplasmic reticulum, Golgi apparatus, endosomes, lysosomes, autophagosomes, and nucleus [20]. Molecular cloning and expression of individual ZIKV proteins reveal distinct and specific organellar localization. ZIKV capsid localizes to several compartments including the nucleus and the Golgi [19]. In addition to capsid, NS2B and NS4A also localize to the Golgi. Three ZIKV proteins namely Env, prM, and NS2A are distributed to the ER as indicated by their co-localization with calreticulin expression. NS5, which contains nuclear localization signals, forms punctate distribution in the nucleus [19]. However, these data should be interpreted with caution as the subcellular localization of these cloned viral proteins may differ in ZIKV-infected cells. For example, individual expression of ZIKV NS3 localizes to the mitochondria, but NS3 is instead localized at the ER when co-expressed with NS2B [21]. Similarly, although NS5 protein is primarily detected in the nucleus of ZIKV-infected cells [22], ZIKV NS5-RNA polymerase has been shown to interact with ZIKV NS3-helicase during viral RNA replication in the ER [23]. Inhibiting this interaction reduces NS3-helicase activity. Subcellular localization of viral proteins is affected by viral proteins interaction, which is crucial for productive infection. Importantly, understanding the mechanisms of viral protein transport and viral protein-protein interaction may provide novel therapeutic targets.

The subcellular distribution of certain ZIKV proteins was corroborated in a separate study where interatomic analyses using proximity-dependent biotin-identification (BioID) labeling and FLAG-based immunoprecipitation (IP) coupled with mass spectrometry (MS) uncover in-depth molecular interactions between ZIKV proteins with various host organelles and proteins. In general, ZIKV proteins mainly interact with host proteins that are involved in protein processing, vesicle trafficking, RNA processing, and lipid metabolism [20]. ZIKV capsid interacts with multiple nucleolar proteins, whereas NS5 protein targets and disrupts the Cajal bodies in the nucleus [20]. This observation is consistent with the aforementioned experimental study that showed nuclear localization of ZIKV capsid and NS5 protein [19]. The study also identified numerous interactions between viral proteins (prM, Env, NS2A, NS2B, NS4A, and NS4B) with various ER proteins, which highlights the importance of the ER in ZIKV life cycle [20].

ZIKV has been reported to be dependent on several ER proteins including ER-associated signal peptidase complex (SPC) proteins, ER translocon, and ER membrane complex (EMC) proteins. SPC, especially SPC subunit 1 (SPCS1), is crucial for ZIKV pathogenesis as knocking out SPCS1 in 293 T cells significantly reduced ZIKV infection and drastically impaired the production of infectious ZIKV particles [24]. A study using pooled CRISPR/Cas9 cell survival enrichment assay identified ZIKV strongly depends on ER membrane complex (EMC) during early-stage ZIKV replication [25]. EMC is a highly conserved oligomeric complex residing on the ER membrane and is crucial for transmembrane protein folding and lipid trafficking [25]. The dependency on six EMC subunits (EMC1-EMC6) was verified with siRNA assays, where depletion of these proteins significantly impaired the replication of several ZIKV strains [25]. Another study reported that ZIKV NS4B physically interact with EMC1 subunit and depletion of this subunit markedly reduced the level of ZIKV NS4A and NS4B protein [26], indicating that EMC proteins are required for viral protein biogenesis. Further investigation using DENV NS4B identified two marginally hydrophobic domains at the N-terminal of NS4B to be crucial for NS4B dependence on EMC [26]. Since ZIKV NS4B shares moderate sequence identity, high sequence similarity, and similar topology with other *Flaviviruses* [27], it is plausible that the two weak hydrophobic domains of ZIKV NS4B are the specific determinants for ZIKV dependency on EMC proteins. In addition to its direct interaction with viral proteins, EMC proteins also associate with ER Sec61 translocon and oligosaccharyltransferase (OST) complex proteins, both of which are also important for ZIKV infection [24, 25]. Sec61 is a major component of the ER translocon that facilitates the entry of nascent polypeptides into the ER lumen for protein processing. ZIKV dependency on Sec61 translocon was validated in a separate study wherein myolactone treatment, a Sec61α inhibitor, dramatically reduced ZIKV expression [28]. ZIKV replication was restored in cells expressing mutant Sec61α that conferred resistance against myolactone inhibition [28]. ZIKV dependency on OST complex, an integral part of the translocon consisting of eight ER-transmembrane protein subunits that catalyzes co-translational N-glycosylation, is based on EMC1, EMC2, EMC4 and EMC5 interaction with OST complex subunits namely STT3A/B, RPN1/2, and DDOST [25]. Additionally, ZIKV proteins including NS4B have been reported to directly interact with these OST subunits [29]. ZIKV dependency on OST complex is corroborated by Marceau et al. that reported a significant abrogation of ZIKV RNA replication in *STT3A* knockout cells [30]. Importantly, treatment with NGI-1, a small molecule OST complex inhibitor, significantly reduced ZIKV RNA replication with an EC_50_ value of 2.2 μM [31]. The inhibitory activity of this non-toxic molecule (CC_50_ = 34.9 μM) is independent of N-linked glycosylation activity of the OST complex as ZIKV replication was drastically impaired in HAP1 cells containing wild-type or catalytic-inactive STT3A [31]. The specific mechanism of ZIKV dependency on STT3A is unclear, but immunoprecipitation and electron microscopy studies showed physical interactions between DENV proteins and STT3A/B, forming viral RNA replication complexes that reside within the ER in close proximity to DENV2-vesicle packets [30]. Since ZIKV proteins also directly interact with STT3A and ZIKV infection induces the formation of vesicle packets [29, 32], it is plausible that ZIKV depends on OST complex in a similar manner.

In short, ZIKV proteins interact with various host proteins and exploit their functions to accommodate the progression of the viral life cycle. ER and ER-related proteins are especially important in the virus genome replication, which may be useful in developing antiviral therapies. The following sections of this review will discuss the structural and molecular changes in the ER following ZIKV infection.

## ZIKV modulates ER structure

Throughout the course of infection, viruses induce various modifications on the host cells’ metabolisms and their cytoarchitecture to support the progression of virus life cycle. One of the common targets is the ER [33]. Among the important functions of the ER include protein processing, synthesis of essential biosynthetic compounds (primarily lipids and proteins), metabolism of steroids and carbohydrates, and storage of calcium ions (Ca^2+^) for intracellular signaling [34]. Structurally, the ER is made of a continuous network of membrane-enclosed flattened sacs and tubules. The dynamic and fluid nature of these structures enable this organelle to accomplish tubule extension, membrane curvature and shape alteration in response to stress-induced or factor-demanding conditions such as cell division and differentiation [34]. Likewise, ZIKV infection is also capable of exploiting the organelle’s plasticity and remodels the ER structure for their replicative benefit.

Recent transmission electron microscopy analyses on ZIKV-infected cells frequently observed significant expansion of the ER [32, 35, 36]. This structural modification typically consisted of proliferating ER lamellae and dilated ER lumen, which cumulatively increases the overall ER size and volume. Enlargement of the ER is believed to occur in response to virus-induced ER stress, due to a supply-demand imbalance that will be elaborated in the next section.

**Fig. 1:**
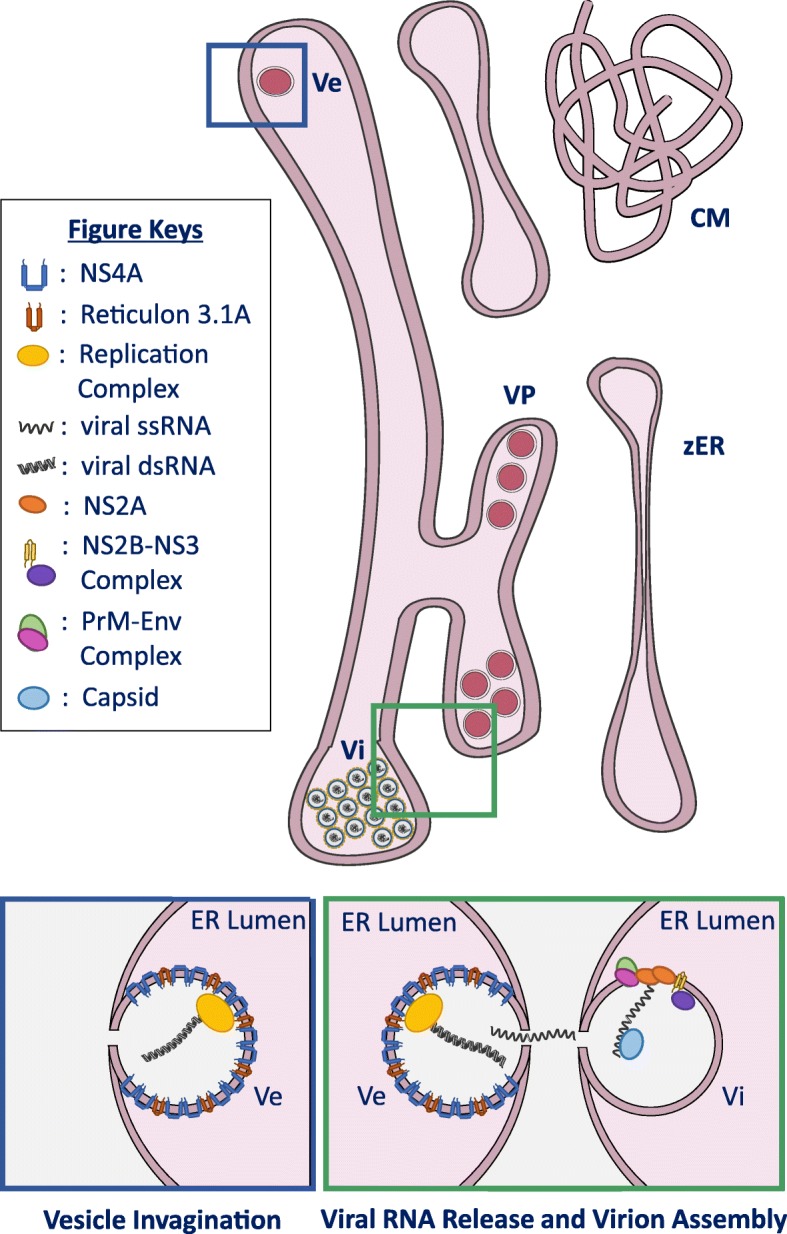
ZIKV infection induces remodeling of the ER structure. ZIKV exploits the ER dynamic characteristic and remodels the ER structure to generate virus-induced structures, vesicles (Ve), vesicle packet (VP), convoluted membrane (CM) and zippered ER (zER), for the benefit of virus replication. Blue and green box depicts schematic representation of host and viral factors involved in vesicle and virus particle (Vi) formation, respectively. ZIKV NS4A utilizes the host reticulon 3.1A (RTN3.1A) to facilitate membrane curvature during vesicle invagination into the ER lumen (blue box). Viral genome replication takes place within this vesicle. Neosynthesized viral RNA genome is released into the cytosol and could undergo either translation, virus particle assembly, or another round of genome replication. Virus particle assembly takes place in apposed ER leaflet. Separate ZIKV NS2A independently recruits viral genome RNA, NS2B-NS3, and unprocessed C-prM-Env complexes, and subsequently congregates at the virus particle assembly site through NS2A oligomerization (green box). Once assembled, NS2B-NS3 proteolytical activity cleaves the recruited C-prM-Env complex to generate individual capsid, prM and Env protein. Cleaved capsid protein interacts with capsid-dense lipid droplets and viral RNA to generate nucleocapsid core followed by Env and prM proteins encapsulation of the nucleocapsid core

ZIKV infection also forms distinct aggregates of intricate ER tubular network that resembles sponge-like matrix called the convoluted membranes (CM) (Fig. 1) [32, 35–37]. CM aggregates are frequently seen bordering ZIKV virions and other ZIKV-induced structures, with occasional virions also found within the CM itself [32, 36]. The function of the CM in ZIKV infection is unclear; nevertheless, evidence from other flaviviruses studies suggest that the CM possesses roles closely associated to protein synthesis and processing [18]. Interestingly, CM aggregates are not observed in ZIKV-infected neural progenitor cells, suggesting cell-type-specific factors are required to develop the CM [32]. No discernible difference in the CM structure is observed between the African and Asian ZIKV strains [36, 37].

Next, vesicle packets (VPs) are also commonly observed in ZIKV-infected cells (Fig. 1) [32, 35–37]. VPs refers to an aggregate of closely packed single-membrane vesicles that are encapsulated within an ER cisterna. Individually, these vesicles measure between 60 and 100 nm in diameter with a narrow channel of approximately 10 nm that connects the vesicle lumen and the cytosol [32, 35]. The radial dimension of these vesicles varies according to the host cell type, thereby hinting possible involvement of cell-type-specific factors in its formation [36]. Modest radial difference was also noted between ZIKV lineages [32]; however, more morphologically pronounced difference between the ZIKV lineages is observed in the vesicle’s shape whereby African strain tend to form ovoid vesicles, whereas the Asian strain’s vesicles are generally more spherical [37]. Nevertheless, these differences, especially the latter, remain contentious due to limited experimental measurements and warrants additional investigations across various cell types to support these observations. Despite these differences, the vesicles’ pore maintain a comparable diameter of 10 nm regardless of the virus strain and the host cell type, suggesting that a conserved cellular or viral machinery mediates pore formation [32].

Invagination of ZIKV-induced vesicles into the ER lumen is mediated through the host reticulon 3.1A (RTN 3.1A) that facilitates ER membrane curvature (Fig. 1, blue box) [38]. Silencing of RTN3.1A impairs NS4A protein stability and results in significant reduction of virus-induced vesicles and virus replication [38]. Typically, these vesicles contain the virus’ replication complex and therefore carries out the virus RNA synthesis or viral replication [32, 36]. Following viral replication, neosynthesized viral genome RNA is released into the cytosol through the vesicle’s narrow channel, where it would consequently either undergo another round of genome replication, translation or virion assembly [17, 30]. In the latter case, ZIKV virion assembles and buds into the ER lumen directly apposed the narrow pore of the replication vesicle (Fig. 1, green box) [32].

Although detailed mechanistic insights of virion assembly remains uncertain, recent study identified that ZIKV NS2A independently recruits viral (+)-ssRNA, NS2B-NS3, and unprocessed C-prM-Env complexes to the virus particle assembly site, possibly through NS2A oligomerization [39]. The same study postulates that virus particle assembly proceeds through NS2B-NS3 protease complex cleaving the recruited C-prM-Env complex. Cleaved capsid protein form nucleocapsid core with the viral RNA and capsid-dense lipid droplet; and subsequently, it is encapsulated by prM and Env proteins, which leads to eventual virion budding into the ER lumen [39]. Ultrastructural analyses within the dilated ER lumen consistently identified virus particles arranged in a two-dimensional paracrystalline array [32, 37]. This array is located in close proximity to the replication vesicles and is therefore largely comprised of fully assembled virions and viral-like particles (enveloped virions without viral genome) [37].

Moreover, ZIKV-infected Huh7 cells also exhibited a unique structural alteration of collapsed ER cisterna or more commonly known as zippered ER (zER) (Fig. 1) that reminisce the observation reported in avian infectious bronchitis virus (IBV) infection, a (+)-ssRNA virus from the Coronaviridae family [32, 40]. This finding presents the first identification of such structure among flavivirus-infected cells and has only been observed in ZIKV-infected Huh7 cells, suggesting that cell-type-specific factors are possibly required to generate this structure [32]. The role of zER in ZIKV infection is unclear; however, zER are frequently found adjacent to VPs suggestive of functions associated with vesicles formation, which is consistent with observations made in IBV-infected cells [32, 40]. Nonetheless, this unique finding warrants further research to elucidate the mechanistic details and biological relevance of the zER.

In addition to the direct modulation of the ER structure, ZIKV also induces the formation of viroplasm-like structures, which is incidentally the first reported observation of such structure in flavivirus infection [41]. Contrary to other ZIKV-induced structures, viroplasms are cytoplasmic inclusion of viral replication components and various relevant host factors associated with virus replication that are formed through reorganization of cell membrane or cytoskeletal elements [42]. These structures are significantly larger than VPs and can measure up to 500 nm in diameter [41]. Viroplasms are primarily located in the perinuclear region and in close proximity to the ER, mitochondria, and microtubules to facilitate viral genome replication [41].

The spatial segregation of virus-induced enclosed structures (viroplasms and vesicles) is postulated to create a subcellular microenvironment concentrated with factors required for virus genome replication, and simultaneously provides physical barriers against RNA nucleases and the host immune responses [32, 42]. The latter, in particular, is consistent with an investigation on WNV that virus-induced structures confer protection, albeit partially, against the host immune mechanism [43].

In summary, ZIKV and several ZIKV proteins localize to the ER and eventually leads to the remodeling of the reticular architecture and exploitation of the organelle’s unique characteristics. These modifications create an ideal environment for viral genome replication, but simultaneously introduce additional burden and stress on the host cell leading to the activation of several cellular responses, which will be discussed in the following sections.

## ZIKV induces ER stress and the unfolded protein response

One of the fundamental roles of the ER is the folding of secreted and membrane proteins, which depends on a multitude of factors including supply of ATPs, stable Ca^2+^ concentration, and a balanced redox environment [44]. Disruption of this specialized environment within the ER, whether through glucose deprivation or viral infection, may lead to overwhelming accumulation of misfolded and unfolded proteins—a condition described as ER stress [45].

Under normal conditions, glucose regulated protein 78 (GRP78), one of the ER molecular chaperones, binds to ER stress sensor transmembrane proteins: protein kinase RNA-activated (PKR)-like ER resident kinase (PERK), activating transcription factor 6 (ATF6), and inositol-requiring enzyme 1 (IRE1). In the presence of misfolded or unfolded protein, GRP78 will bind to exposed hydrophobic residues of these proteins to induce proper protein folding [45, 46]. Accordingly, during ER stress, the accumulation of misfolded or unfolded proteins saturates the free pool of GRP78 and titrates the bound GRP78 away from the stress sensors leading to activation of respective sensor’s molecular pathways [45]. These molecular initiatives and their corresponding downstream effects are collectively known as the unfolded protein response (UPR). This evolutionarily conserved countermeasure induces pro-survival responses including global arrest of protein synthesis, upregulation of protein degradation factors, and enhanced protein folding capability [45]. However, under severe ER stress conditions, UPR will instead trigger apoptosis.

**Fig. 2:**
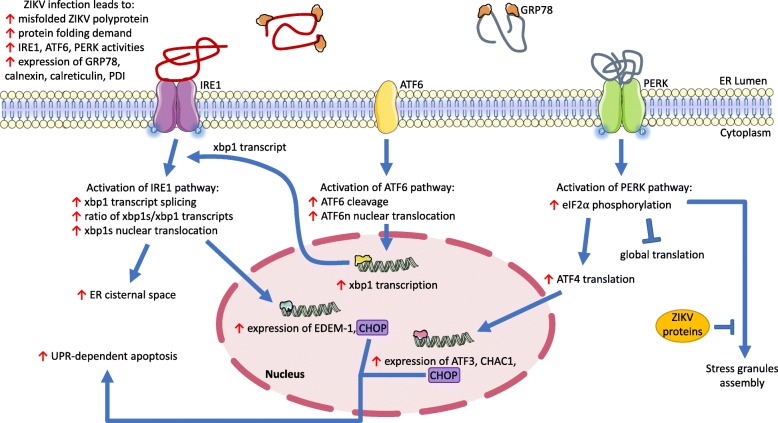
ZIKV-induced ER stress initiates host cell unfolded protein response (UPR). ZIKV infection induces ER stress due to the increased amount of unfolded/misfolded viral (red strand) and host cell (grey strand) protein aggregates in the ER lumen. The accumulation of these partially processed proteins leads to the overwhelming demand of correct protein folding. To facilitate protein folding, GRP78 (orange) dissociates from the stress sensors (IRE1, ATF6, and PERK) and binds to the misfolded/unfolded proteins. Expression of other chaperones is also elevated to address the overwhelming protein folding demand during ZIKV infection. Binding of misfolded protein to PERK (green) activates the sensor and triggers phosphorylation of eIF2⍺ that in turn stimulates 1) global translational block except for selective mRNA involved in UPR, and 2) formation of stress granules. However, ZIKV bypasses global translation block and inhibits stress granules formation, but the mechanistic details of these viral interference are still unclear. Activation of ATF6 (yellow) promotes the protein proteolytical processing in the Golgi apparatus into ATF6n, ATF6n nuclear translocation, and expression of UPR target genes including *XBP1*. Activated IRE1 (purple) splices *XBP1* transcript which produces an active transcription factor (XBP1s) that stimulates expansion of the ER volume, and expression of CHOP and ERAD factors such as EDEM-1. Alternatively, IRE1 can trigger ASK1-p38 MAPK pathway that enhances CHOP apoptotic activities and promotes other apoptotic-related activities under severe ER stress condition. Blue pointed arrows denote activation, blue blunt-end arrows denote inhibition, and red pointed arrows denote increased expression/activity

Following ZIKV infection, the accumulation of misfolded virus polyproteins in the ER lumen overwhelms the ER protein-folding capacity leading to ER stress and triggers the activation of the UPR (Fig. 2) [47]. Additional evidence of ER stress and UPR activation are demonstrated in the elevated expression of GRP78 and other chaperones such as calnexin, calreticulin, and protein disulfide isomerase (PDI) in ZIKV-infected neural cells in vitro and in vivo [35, 48, 49]. Increased expression of these ER stress markers was accompanied by impaired indirect neurogenesis and microcephalic phenotype in mice [49]. This finding is consistent with a previous report wherein the induction of UPR in cerebral apical progenitor cells tipped neuronal differentiation towards direct neurogenesis at the expense of indirect neurogenesis, leading to depleted intermediate progenitors, reduced overall cortical neuron output, and diminished cerebral volume, which ultimately caused microcephaly in vivo [50].

In addition to elevating the expression of ER chaperone proteins, ZIKV has been shown to induce UPR by activating the stress sensors as summarized below (Fig. 2).

### ZIKV and ATF6

ATF6 is a type II ER transmembrane protein that contains a transcription activation domain (TAD) on its cytosolic-facing N-terminal and two independent Golgi-localization signals (GLS) on its luminal-facing C-terminal [51]. Following the dissociation of GRP78 that unmasks the GLS, ATF6 translocate to the Golgi apparatus and undergoes sequential proteolytic processing by site-1 and site-2 proteases, liberating TAD-containing ATF6 N-terminal (ATF6n) [51]. Subsequently, ATF6n enters the nucleus and binds to ER stress response elements in the promoter region of UPR-target genes, including X-box binding protein 1 (*XBP1*) transcription factor, a downstream effector of IRE1 stress sensor pathway, and *GRP78* to autoregulate the UPR [51–53].

ZIKV infection has been shown to activate the UPR via ATF6, where ATF6 proteolysis and ATF6n nuclear translocation were induced in vitro and in vivo [48].

### ZIKV and IRE1

IRE1 is a single-pass transmembrane protein. The protein’s ER luminal domain serves as a stress sensor, whereas the cytosolic domain is sub-divided into kinase and ribonuclease (RNase) domains. Under ER stress, GRP78 releases from IRE1 luminal domain and binds onto the misfolded/unfolded proteins. This dissociative event frees the luminal domain and in turn, permits binding of the misfolded/unfolded protein onto the liberated domain [54, 55]. Consequently, IRE1 oligomerizes at its luminal domain, which brings the kinase domains in close proximity leading to auto-phosphorylation of the kinase domains [56]. The phosphorylation event at the activation loop is postulated to induce conformational changes of the RNase domain that permit a more efficient binding of RNA substrates [57]. Activated IRE1 cleaves off 26 nucleotides from *XBP1* transcripts, resulting in a translational frameshift to produce a potent transcriptional activator (XBP1s) that elevates the expression of UPR-target genes to promote protein folding, processing, and secretion [53, 58]. XBP1s also modulates the expression of factors involved in ER-associated protein degradation (ERAD), a cellular process of eliminating aberrant proteins through retro-translocation and proteasomal degradation [45].

Alternatively, IRE1 protein can also mediate ER stress-induced apoptosis following chronic stress damage by triggering activation of apoptotic signaling kinase 1 (ASK1), which consequently activates p38 mitogen-activated protein kinase (p38 MAPK) and Jun N-terminal kinase (JNK) leading to apoptosis [59]. JNK mediates apoptosis primarily by inhibiting the anti-apoptotic B-cell lymphoma 2 (BCL-2) and activating the pro-apoptotic BIM protein [59], whereas p38 MAPK promotes apoptosis by phosphorylating CCAAT-enhancer-binding protein (C/EBP) homologous protein (CHOP) at serine residues 78 and 81 to enhance its transcriptional activity and induce apoptosis [60, 61]. CHOP is expressed downstream of all three ER stress sensors, indicating overlapping UPR pathways during severe ER stress [62]. CHOP forms heterodimers with other C/EBP family transcription factors; therefore, the basic domain in CHOP renders the transcription factors incapable of binding to their respective DNA binding sites, which reduces the expression of their target proteins including BCL-2 [63]. CHOP also contains a TAD and thus, can bind to a unique sequence to induce the expression of target genes including *BIM* [63, 64].

ZIKV infection has been shown to promote *XBP1* splicing and XBP1s translocation into the nucleus, leading to elevated expression of XBP1 downstream effectors such as ER degradation-enhancing α-mannosidase-like 1 (EDEM-1) and CHOP, indicating the activation of the IRE1 arm of the UPR [48]. EDEM-1 is an enzyme involved in the ERAD process that recognizes and facilitates retro-translocation of misfolded proteins to the cytosol for subsequent proteasomal degradation and thus, alleviating stress damage [65]. Instances of aggravated ER stress were also demonstrated in ZIKV-infected cells, which lead to elevated expression of CHOP protein and initiation of ER-stress-induced apoptosis [49]. Pharmacological intervention using IRE1 inhibitor, 4μ8C, prevented microcephaly and impaired ZIKV replication in mouse fetal brains [49]. Modulation of IRE1-ASK1 pathway during ZIKV infection is still unclear.

Previous study reported that ectopic expression of XBP1 promotes expansion of the ER, a visual sign often manifested in response to ER stress [66]. This morphological change is hypothesized to reduce the local concentration of misfolded/unfolded proteins and accommodate the overwhelming demand for protein folding—a significant portion of which is neosynthesized viral polyproteins [47, 67]. Consistent with increased expression of XBP1, ultrastructural characterization of ZIKV-infected cells reported significant ER enlargement extending across the cytoplasm and is related to ZIKV-induced ER stress [32, 35, 36, 47].

### ZIKV and PERK

Similar to IRE1, release of GRP78 proteins enable misfolded/unfolded proteins binding onto exposed PERK luminal domain leading to oligomerization and auto-phosphorylation of PERK protein to activate its downstream pathway [55, 68]. PERK phosphorylation, in turn, induces eIF2α phosphorylation (p-eIF2α) that mediates three interlinked UPR mechanisms: expansion of protein folding capacity, suppression of nascent protein production and induction of apoptosis in the event of severe stress [69]. Phosphorylation of α subunit in the eIF2 complex hinders the assembly of preinitiation complex (PIC) by blocking the activity of eIF2B guanine exchange factor, leading to global suppression of mRNA translation [69]. However, selected mRNAs, typically transcripts for UPR machinery, can bypass this translational block [69]. Interestingly, viruses have evolved various mechanisms to overcome this translational block, but the mechanism of this process in ZIKV infection is unclear. Recent analyses of ZIKV infection in vitro and in vivo reported a substantial increase of eIF2α phosphorylation and elevated expression of several downstream PERK effectors such as *ATF4, ATF3, CHAC1,* and *CHOP* [48, 49]. Importantly, intracerebroventricular administration of pharmacological PERK inhibitor in ZIKV-infected mice restored appropriate neurogenesis balance and rescued infected mouse embryos from microcephalic phenotype. However, unlike IRE1 inhibitor, PERK inhibitor did not affect ZIKV replication [49]. PERK inhibitor also prevented microcephaly in placental ZIKV inoculation model, which mimics natural ZIKV vertical transmission [49].

In summary, ZIKV localizes to the ER for viral genome replication. These additional transcriptional and translational processes impart significant burden on the ER leading to ER stress and UPR induction. During the early stage of UPR, UPR effectors elicit adaptive responses to mitigate ER stress. These responses include the upregulation of chaperones and protein processing enzymes to promote and rectify protein folding, induction of ERAD to degrade misfolded/unfolded proteins, global translational arrest, and activation of autophagy and/or reticulophagy.

## ZIKV subverts other ER stress responses

Beside UPR, ER stress also concomitantly induces several other cellular processes. Specifically, stress granules assembly and ER autophagy impart fundamental importance in regulating translational arrest and ER homeostasis, respectively. ZIKV have been reported to subvert these processes to allow the progression of viral replication.

### ZIKV inhibits cytoplasmic stress granules formation

ER stress signal can be transmitted to other cellular components to rescue cell survival. A clear example to illustrate stress signal transmission is the generation of stress granules (SGs) in the cytoplasm to stall initiation of translation [70]. SGs assembly are generally triggered through two distinct categories of mechanisms: 1) eIF2α-independent and 2) eIF2α-dependent mechanisms [71]. In particular, the latter category, or more specifically eIF2α-phosphorylation, is mediated by PKR, PERK, general control non-derepressible-2 (GCN2), and heme-regulated inhibitor kinase (HRI) in response to diverse cellular stress signals [71]. SGs are ribonucleoprotein (RNP) structures primarily composed of non-translating mRNAs, stalled translation initiation complexes, and RNA-binding proteins [70]. The formation and components of SGs are reviewed in details here [71]. SGs assembly during global translational arrest negatively impact viral genome translation as the SGs reduce the accessibility of translational machinery complexes [72]. Following the relief of translation suppression, the SGs are disassembled via several mechanisms, one of which involves eIF2α dephosphorylation by Growth Arrest and DNA-Damage-inducible 34 (GADD34) protein [73]. This allows the PIC to resume protein translation at the surface of the SGs, resulting in SGs shrinkage and disappearance [71].

Viruses have adopted several mechanisms to repress the assembly of stress granules and utilize SG protein components for viral polyprotein synthesis instead [74]. For example, herpes simplex virus genome encodes γ_1_34.5 protein that functionally mimics the activity of GADD34 and directs the dephosphorylation of eIF2α [75]. Alternatively, coronaviruses repress the assembly of stress granules by asserting an inhibitory effect on PKR activation and upregulating the expression of GADD34 [76]. Likewise, ZIKV also suppresses the formation of stress granules in favor of virus replication by upregulating the expression of GADD34 [77]. Consistent with this finding, pharmacological inhibition of GADD34-mediated eIF2α dephosphorylation rescued SGs assembly and decreased ZIKV particles production [77]. Additionally, another study reported that ZIKV proteins, namely capsid, NS3, NS2B-NS3, and NS4A proteins, suppressed SGs assembly; however, this modulation was observed in an eIF2α-independent manner [78]. ZIKV capsid inhibited SGs assembly by forming stable complexes with SG core proteins, Ras GTPase-activating protein-binding protein 1 (G3BP1) and caprin-1, but not with T-cell-restricted intracellular antigen 1 related (TIAR) protein [78]. ZIKV utilized these SG core proteins for viral protein and virion production. Beside regulating eIF2α phosphorylation and hijacking key SG proteins, RNA viruses were reportedly capable of interfering SGs assembly through cleaving and redistributing SGs nucleating factors [74]. It was previously reported that ZIKV NS2B-NS3 protease could cleave host antiviral factors to impair intrinsic host defense; intriguingly, ZIKV did not mediate the cleavage of SG factors even though SGs assembly was affected by ZIKV NS3 and NS2B-NS3 protease [77–81]. Nonetheless, ZIKV infection was found to facilitate the redistribution of TIAR to viral replication sites, which correlates to viral replication output [77, 78, 81]. Hence, it is unclear how ZIKV NS3, NS2B-NS3, and NS4A proteins block SGs formation and whether these viral proteins affect eIF2α phosphorylation. Interestingly, a recent study also identified a key SG-interacting protein, human antigen R, exhibits substantial anti-ZIKV effect potentially by destabilizing the ZIKV RNA [81].

### ZIKV inhibits reticulophagy

As previously discussed in Section 4, the induction of ER stress initiates several adaptive countermeasures such as upregulation of UPR pro-survival factors and ER enlargement to repair stress-induced damages and restore normal cellular functions. Interestingly, initial studies in mammalian and yeast cells revealed that a fraction of cells also contain autophagosome-like structures following ER stress induction [82, 83]. These autophagic vacuoles expel selectively excised ER membrane containing aberrant protein aggregates [84]. This process, known as reticulophagy, is an additional mechanism that occurs in parallel with UPR to regulate ER volume and homeostasis [85]. Autophagy, including reticulophagy, is also a host innate defense mechanism as these autophagosomes have been shown to incorporate viral proteins for degradation [86].

Reticulophagy, also known as ER-phagy, is mediated by ER-phagy receptors such as Family with Sequence Similarity 134 Member B (FAM134B) and reticulon-3 (RTN3) proteins that reside on the ER membrane. These autophagy receptors sequester ER fragments via its LC3-interacting-region (LIR) domain interaction with autophagosomal-presenting microtubule-associated protein 1 light chain 3 (MAP 1LC3) [87]. Similar to other ER-shaping proteins, FAM134B also possesses a reticulon homology domain (RHD), a motif that promotes membrane curvature [87].

In ZIKV-infected cells, depletion of FAM134B protein renders notable ER expansion and significant upregulation of ZIKV replication activity [80]. This is due to the proteolytic activity of ZIKV NS2B-NS3 that cleaves exposed RHD located on FAM134B protein, which impedes the oligomerization of FAM134B proteins and consequently, prevents ER membrane excision and reticulophagy from taking place [80]. This efficiently blocks host cell innate defense mechanism from degrading ZIKV proteins.

To summarize, ZIKV virus bypasses the UPR by inhibiting stress granules assembly and reticulophagy to ensure continuous viral protein translation and virion production while simultaneously protecting the virus from host cell defense mechanisms. Prolonged ER stress exacerbates the stress condition and leads to the activation of another arm of the UPR: cell death.

## ZIKV-induced ER stress leads to paraptosis-like cell death

Countless studies showed that ZIKV infection causes cell death through several cell death mechanisms such as paraptosis and apoptosis [12, 88] Apoptotic events in ZIKV-infected cells occur through both the intrinsic and extrinsic pathways based on the activation of pathway-specific caspase-9 and caspase-8, respectively [88, 89]. Interestingly, ZIKV Env protein is sufficiently capable to induce pro-apoptotic expression profile that represents intrinsic apoptosis including elevated expression of tumor protein p53, which correspondingly mediates the expression and activity of its downstream targets by suppressing anti-apoptotic BCL-2 and facilitating expression of pro-apoptotic Bcl-2-associated X (BAX) [90]. This modulation consequently reduces mitochondrial membrane permeability; thus, aiding the release of cytochrome c, formation of apoptosome, and initiation of caspase cascade, which eventually leads to cell death [62]. Additionally, increased level of several apoptotic markers, such as tumor necrosis factor alpha and receptor-interacting protein 1, in ZIKV-positive microcephalic neural specimens are suggestive of extrinsic apoptotic activation [91].

As previously mentioned, ER stress elevated the expression of *CHOP* to initiate apoptosis in ZIKV-infected cells [48, 49]. Prolonged ER stress could also trigger non-apoptotic cell death as demonstrated by an investigation using time-lapse and electron microscopy that revealed the formation of extensive ER-derived vacuoles within the cytoplasm of ZIKV-infected cells, which eventually resulted in paraptosis-like death [28]. This observation is consistent with earlier report that showed cytoplasmic vacuolization in ZIKV-infected human skin biopsy specimens [13]. Vacuole formation was inhibited with class I PI3K/Akt inhibitor treatment. More importantly, pan-caspase inhibitor, ZVAD-FMK, only slightly rescued cell survival but did not affect vacuolization [28], implying that certain ZIKV strains, namely HD78788, PF13 and NC14, induce cell death primarily through paraptosis-like death. The role of PI3K/Akt pathway in ZIKV-induced cell death was verified by a recent study that reported treatment with AR-12, a celecoxib derivative kinase inhibitor, significantly inhibited the replication of ZIKV in A129 mice and improved mice survival mainly through Akt down-regulation [92].

## Conclusion

**Fig. 3:**
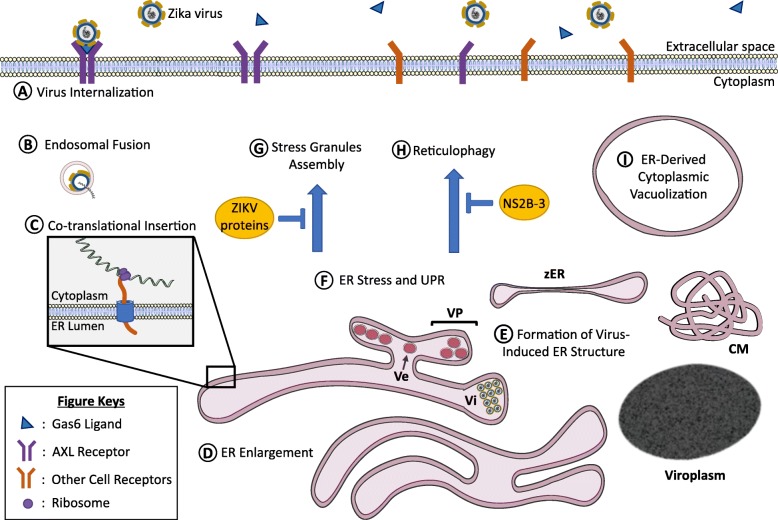
ZIKV proteins localize to the endoplasmic reticulum (ER) and cause molecular and structural changes. **a**) Zika virus (ZIKV) indirectly interacts with AXL receptor and internalizes into the host cells through clathrin-mediated endocytosis. Other cell receptors such as DC-SIGN can also serve as ZIKV entry receptor. **b**) Acidic endosomal microenvironment facilitates endosomal fusion thereby, releasing ZIKV RNA genome that is immediately bound onto cytosolic ribosomes. **c**) Translation of the viral polyprotein (orange) likely initiates in the cytosol and continues with co-translational insertion into the ER membrane via Sec61 translocon complex (blue). Post-translational processed ZIKV proteins induce ER structural alterations such as **d**) enlargement of the ER, and **e**) formation of convoluted membrane (CM), vesicle packets (VPs), zippered ER (zER) and viroplasms. Simultaneously, accumulation of misfolded and unfolded ZIKV proteins in the ER lumen results in **f**) the induction of ER stress and thus, initiates the host unfolded protein response (UPR) and other intrinsic defense mechanisms including reticulophagy and stress granule formation. ZIKV proteins **g**) impede formation of stress granules and **h**) inhibit reticulophagy to sustain viral replication. ZIKV infection also induces **i**) ER-derived cytoplasmic vacuolization, a visual characteristic of paraptosis. Blue pointed arrows denote activation, blue blunt-end arrows denote inhibition. Ve, virus-induced vesicle; Vi, virus particle

ZIKV, like other viruses, is an intracellular parasite that largely depends on the host biosynthetic, energetic and structural resources for successful viral replication and propagation. In particular, exploitation of these resources is manifested through the manipulation of the endoplasmic reticulum architecture and the processes that take place within or in proximity to the ER as summarized in Fig. 3. Briefly, ZIKV infection leads to structural changes of the ER such as ER enlargement due to the accumulation of misfolded/unfolded ZIKV proteins (Fig. 3d) and the formation of convoluted membranes, vesicle packets, zippered ER, and viroplasm-like structures for viral RNA replication (Fig. 3e). The accumulation of misfolded/unfolded proteins induce ER stress and triggers the UPR (Fig. 3f). In parallel, ER stress also initiate several response mechanisms such as stress granules assembly to impede global protein translation (Fig. 3g) and reticulophagy to remove damaged ER (Fig. 3h). However, ZIKV are able to bypass these processes through various strategies, which eventually lead to paraptosis-like cell death (Fig. 3i).

Although the number of ZIKV cases have subsided, ZIKV remains a significant threat due to the sporadic and unpredictable nature of its outbreak. In addition, ZIKV shares the same vectors with another widespread flavivirus, DENV [93], which is projected to increase exponentially in the future [94]. Thus, the potential re-occurrence of outbreaks, coupled with devastating neurological complications, warrants for extensive research to completely understand the virus pathophysiology for antiviral drug development. This is effectively demonstrated by several studies included in this review that employ multiple omics technologies to identify and target ER-associated ZIKV dependency factors and ER stress sensors. This strategy could pave the way in developing anti-ZIKV drugs.

## Data Availability

Not applicable.

## Abbreviations

ATF6: Activating transcription factor 6
CM: Convoluted membrane
eIF2: Eukaryotic initiation factor 2
EMC: ER membrane complex
ER: Endoplasmic reticulum
GRP78: Glucose-regulated protein 78
IRE1: Inositol-requiring enzyme 1
NS: Non-structural
PERK: Protein kinase RNA-activated (PKR)-like ER resident kinase
SGs: Stress granules
SPC: Signal peptidase complex
UPR: Unfolded protein response
VPs: Vesicle packets
XBP-1: x-box binding protein 1
zER: zippered ER
ZIKV: Zika virus
